# Insights on Aortoenteric Fistulas from a Tertiary Referral Center: Diagnostic Algorithm, Management and Outcomes

**DOI:** 10.3390/medsci14050578

**Published:** 2026-09-17

**Authors:** Hassan Al-Thani, Khalid Ahmed, Ayman El-Menyar, Ahmed Sadek, Ahmed Hussein, Eman Elmenyar, Hazem El Beyrouti, Abdulwahid Almulla

**Affiliations:** 1 Department of Surgery, Vascular Surgery, Hamad Medical Corporation, Doha P.O. Box 3050, Qatar; halthani@hamad.qa (H.A.-T.); amohamed4@hamad.qa (A.S.); aelnaby@hamad.qa (A.H.); hbeyrouti@gmail.com (H.E.B.); 2 Department of Surgery, Trauma Surgery, Hamad Medical Corporation, Doha P.O. Box 3050, Qatar; kahmed11@hamad.qa; 3 Department of Internal Medicine, Weill Cornell Medical College, Doha P.O. Box 24144, Qatar; 4 Clinical Research, Trauma and Vascular Surgery, Hamad Medical Corporation, Doha P.O. Box 3050, Qatar; 5 Faculty of Medicine, Bahçeşehir University, 34353 Istanbul, Turkey; eelmenyar@gmail.com; 6 Department of Cardiac and Vascular Surgery, University Medical Centre Mainz, Johannes Gutenberg University, 55131 Mainz, Germany; 7 Department of Cardiac Surgery, Hamad Medical Corporation, Doha P.O. Box 3050, Qatar; aalmulla@hamad.qa

**Keywords:** aortoenteric fistula, endovascular aortic repair, stenting, open aortic surgery, aortoesophageal, aortoduodenal, GIT surgery

## Abstract

**Background**: Aortoenteric fistulas (AEFs) are uncommon emergency surgical conditions associated with high mortality. We explored the presentation and management of AEFs in a tertiary hospital in Qatar, a rapidly developing Middle Eastern country. By highlighting our institutional experience with ADFs and AOFs, we aim to contribute to the growing body of literature on this rare condition and to emphasize the importance of early diagnosis, appropriate surgical planning, and multidisciplinary care in optimizing patient outcomes. We also proposed a diagnostic classification of AEFs to better understand their location, relationship to the aorta, and pathology on both sides (the gastrointestinal tract and Aorta) for further studies. **Methods**: This was an observational, descriptive, retrospective, single-center case series that included 10 consecutive cases presenting with AEFs. We reviewed the clinical presentation, surgical management, and outcomes of AEF cases from 2008 to 2024. Data were extracted from electronic medical records and operative notes. Additionally, we reviewed the literature over the last two decades to summarize recently published English-language literature on aortoenteric fistulas (types, presentations, management and outcomes). **Results**: This observational case series included nine males and one female; four with aortoesophageal (AOF) and six with aortoduodenal (ADF) fistulas. The main presentations were hypotension and upper gastrointestinal bleeding. Blood cultures were positive in four cases, including Escherichia coli, Candida albicans, and Klebsiella. Foreign bodies were the underlying cause in two cases, while an old aortic coarctation repair was found in one case. Surgical repair was not completed in two patients who deteriorated quickly and died with hypovolemic shock. Two patients had thoracic endovascular aortic repair, and others had open surgeries. The overall AEF-related mortality was 60% (*n* = 6; open surgery group). **Conclusions**: This case series could support the importance of prompt diagnosis and multidisciplinary management. It also underscores the importance of individualized treatment to optimize survival. The proposed diagnostic algorithm still requires further evaluation and validation within large samples before its implementation on a large scale.

## 1. Introduction

Aortoenteric fistula (AEF) is a rare, life-threatening condition resulting from an abnormal connection/fistula between the aorta and gastrointestinal tract [1]. Despite high morbidity and mortality, especially in open surgical cases, selected patients managed with endovascular therapy demonstrate favorable long-term outcomes. AEFs are associated with life-threatening hemorrhage and sepsis, making it a vascular emergency with high mortality if not promptly diagnosed and treated [2,3].

AEFs are classified as primary, which often occur due to aneurysmal erosion into the adjacent gastrointestinal tract (GIT), or secondary, arising as a complication following aortic reconstructive surgery or endovascular aortic repair (EVAR) [4]. Primary AEFs are due to aortic aneurysm or vasculitis, while secondary AEFs are due to prior aortic surgery. Primary AEFs have an incidence rate of 0.007 per million [1,5]. Secondary AEFs are more common, with a rate of 0.36% to 1.6% and typically result from aortic graft infection, erosion, or mechanical pressure on adjacent intestinal structures [6,7,8].

In gastrointestinal oncology, the occurrence of an AEF is rare, and when it occurs, it is typically through tumor invasion, advanced local disease, postoperative or post-radiation changes weakening tissue planes.

A systematic review showed that the underlying pathology of AEFs originates in the abdominal aorta (56.1%) and thoracic aorta (43.9%). AEFs involving the abdominal aorta are mainly secondary fistulas (78%), whereas the thoracic AEFs are mostly primary type (72%), and 16% of AEFs reported are due to GIT malignancy [8]. The clinical presentation of AEFs is variable and often nonspecific, posing a diagnostic challenge. Patients may present with transient GIT bleeding known as ‘herald bleed’, seen in 52–75% of patients, which signifies impending massive hemorrhage [9]. Additionally, abdominal or chest pain, abdominal pulsation, sepsis, and signs of shock are possible presentations of AEFs [10,11,12].

AEFs are also named according to the site of communication between the aorta and GIT parts, i.e., esophagus (aortoesophageal; AOF), and duodenum (aortoduodenal; ADF). In a recent review, aortoesophageal fistulas (AOF) occur due to aneurysm (54.2%), foreign body (19.2%) and esophageal cancer (17%) [7]. The duodenum is the most frequently involved segment of the GIT, particularly in secondary AEFs [13]. The AOF, although less common, are typically associated with thoracic aortic disease or foreign body ingestion and carries a poor prognosis due to mediastinal contamination and difficulty with surgical access [14]. The AOF has a classic presentation known as Chiari’s triad, which includes mid-thoracic dysphagia, herald bleed, followed by hemorrhagic shock [15,16,17,18].

Timely diagnosis requires a high index of suspicion, especially in patients who have a history of aortic surgery presenting with GI bleeding or sepsis. Contrast-enhanced computed tomography and endoscopy are cornerstone diagnostic tools, though findings may be subtle or transient [19,20].

Surgical management remains the gold standard, including open repair with graft excision and reconstruction, endovascular techniques (TEVAR or EVAR), or hybrid approaches [21]. Patient stability, anatomical considerations, and the presence of infection influence the choice of technique. Despite advances in surgical and critical care, AEFs continue to carry high perioperative and long-term mortality rates [22].

This case series presents the clinical characteristics, microbiological findings, surgical management, and outcomes of ten patients diagnosed with AEFs. By highlighting our institutional experience with ADFs and AOFs, we aim to contribute to the growing body of literature on this rare condition and to emphasize the importance of early diagnosis, appropriate surgical planning, and multidisciplinary care in optimizing patient outcomes.

## 2. Materials and Methods

This was an observational, descriptive, retrospective, single-center case series that included 10 consecutive cases presenting with AEFs at Hamad General Hospital (HGH) between 2008 and 2024. This is the only tertiary non-profit governmental hospital in the country that manages vascular injuries through open surgery, endovascular, or medical options. Qatar is a rapidly developing Middle Eastern country.

We extracted data from the electronic medical records (EMAR) and operative notes to identify all patients with AEFs who were treated at our institution during the study period. The diagnosis of AEF was made according to clinical evaluation, CT criteria and endoscopy. AEFs were classified anatomically as AOFs or ADFs, and etiologically as primary or secondary. A primary AEF was defined as a pathologic communication involving the aorta without previous aortic reconstruction; a secondary AEF was defined as a fistula arising as a complication following prior open or endovascular aortic repair. The inclusion criteria were patients who presented with AEF, diagnosed clinically and radiographically, regardless of sex, age, or race. Data were extracted from the electronic medical record and operative notes. All data were kept coded and confidential. Age was given as a range in groups for de-identification: young (1) 30–40 and (2) 41–54, and old (1) 55–65 and (2) 66–80 years.

There is a multidisciplinary team for AEF management, including a general surgeon, an infectious disease physician, a cardiac surgeon, and a vascular surgeon.

This study was approved by the Research Ethics Committee of the Medical Research Center (MRC) at Hamad Medical Corporation (MRC-04-25-1077 on 24 September 2025). No direct contact with participants, and data were collected anonymously and retrospectively. There are no identifiers, including the photo, that should be in the manuscript. GenAI was not used to generate text, data or graphics or assist in study design or data collection, analysis or interpretation.

Statistical analysis for basic descriptive statistics such as median, range, percentages, and averages was conducted whenever applicable. Kaplan–Meier survival curve was made describing the pattern of survival and censored cases. All data analyses were carried out using SPSS version 22 (Armonk, NY, USA). Additionally, we reviewed the literature over the last two decades to summarize recently published English-language literature on aortoenteric fistulas (types, presentations, management and outcomes). Moreover, we proposed a diagnostic algorithm for better classification, highlighting the importance of considering both individual and combined pathological changes on both sides (the aorta and the GIT). This will help decision-makers with proper management after being validated in larger samples and multicenter studies before use in further studies.

## 3. Results

This retrospective case series included 10 patients (nine males and one female). The mean age was 58 years (range, 30–80 years) for males, and the female was 30–40 years old. We applied the proposed diagnostic algorithm to the 10 cases, as shown in Table 1. Detailed lesions and interventions are presented in Table 2 (AOFs) and Table 3 (ADFs).

Causes of AEFs: A foreign body was suspected in two cases as a reason for the fistula, while an old aortic coarctation repair was found in one case. Surgical repair was not completed in two patients who deteriorated quickly and died with hypovolemic shock. Two AOFs secondary to foreign bodies (one fishbone and one chicken bone) both presented with massive GI bleed; one had cardiac arrest post-endoscopy before any surgical intervention; the second one had a cardiac arrest in the operating room after thoracotomy; both died. Four primary ADFs and six secondary fistulae (two AOFs and two ADFs).

**Figure 1:**
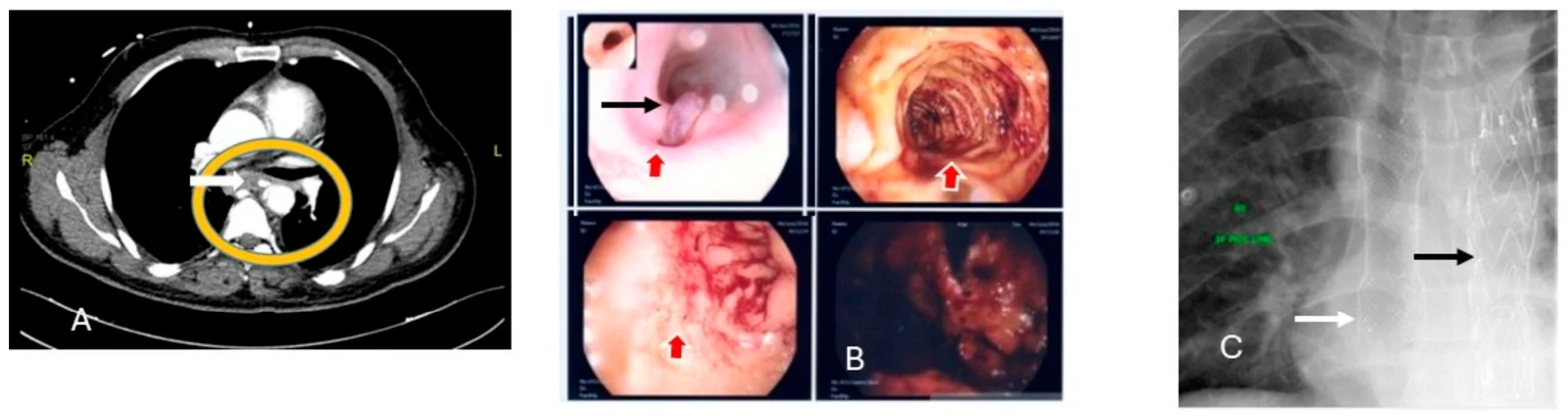
(Case 3): (**A**) CT scan with contrast, showing large contrast extravasation into the esophagus, indicating AEF (circle and white arrow). (**B**) Picture taken for upper endoscopy. In the lower esophagus, it shows a clot protruding (black arrow) through an ulcer (red arrow) and pointing down towards the distal esophagus. The other pictures of the stomach show blood within the stomach (red arrow) and duodenum (red arrow). (**C**) Chest X-ray showing the EVAR stent (black arrow) and esophageal stent (white arrow).

**Figure 2:**
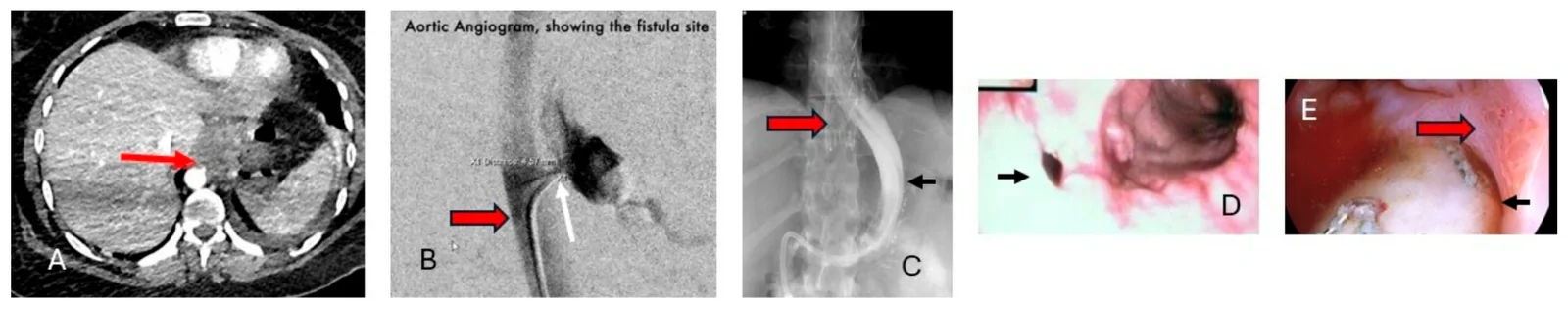
Case 4: (**A**) CT scan with contrast, showing contrast extravasation indicating AEF (red arrow indicate the site of fistula). (**B**) Aortogram showing the thoracic aorta and the angio catheter (red arrow) contrast leaking into the esophagus (white arrow). (**C**) Oral contrast shows no leak or fistulas following the TEVAR stent placement (black arrow). (**D**) Upper endoscopy shows ulcer in the lower esophagus (black arrow). (**E**) Follow-up endoscopy revealed complete erosion of the vascular stent (black arrow) into the esophagus (red arrow) when the patient presented with upper GI bleeding.

**Figure 3:**
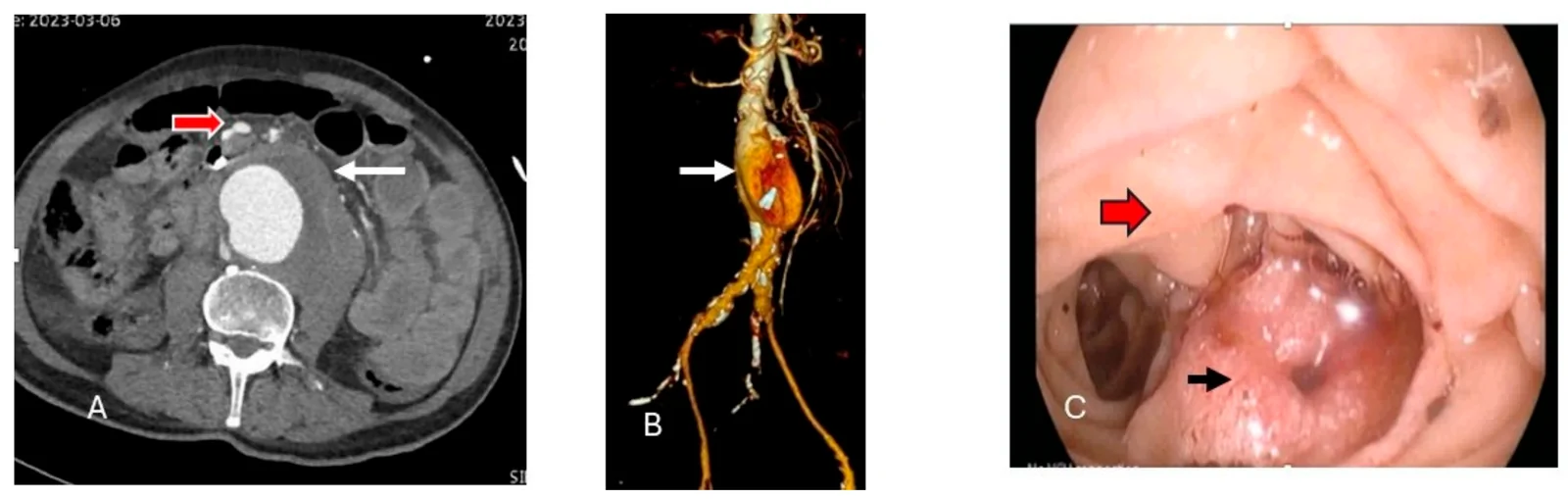
(Case 5): (**A**) CT scan with contrast (white arrow), indicating a large aneurysm with contrast in the small intestine (red arrow). (**B**) Reconstructed CT scan with contrast (white arrow), indicating the large infrarenal abdominal aortic aneurysm. (**C**) Endoscopy of the fourth part of the duodenum (red arrow) showing an ulcerating lesion with blood on the base of the ulcer (black arrow); it was actively bleeding during the endoscopy.

**Figure 4:**
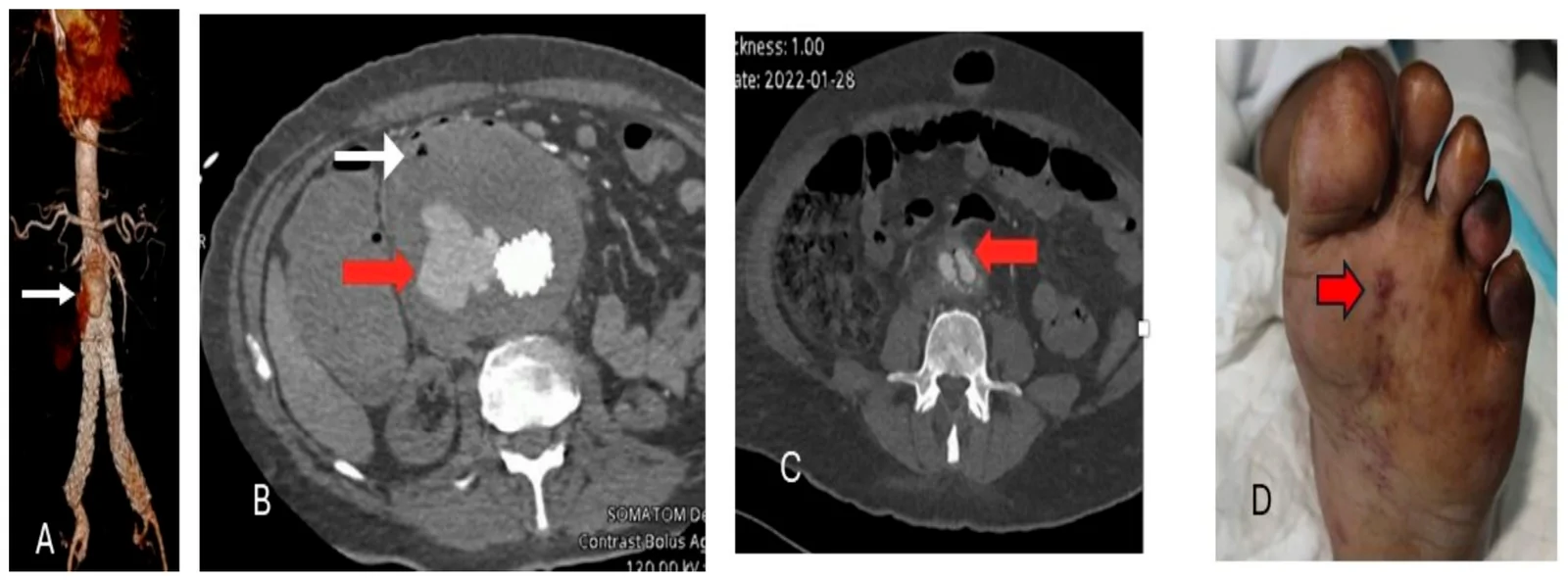
(Case 7): (**A**) Reconstructed CT scan with contrast, white arrow indicating large aneurysm with type one endoleak, also showing EVAR (Endovascular Aneurysm Repair) stent–graft. (**B**) CT scan with contrast (red arrow), indicating the large infrarenal abdominal aortic aneurysm with contrast outside the graft, indicating type I endoleak, and with an air bubble within the sac of the abdominal aneurysm, indicating AEF (white arrow). (**C**) Reconstructed CT scan with contrast (red arrow), indicating the distal part of the bifurcated graft and contrast; there is air around the graft, indicating infection. (**D**) A photo of the left foot showing ischemic changes (red arrow) due to septic emboli.

**Figure 5:**
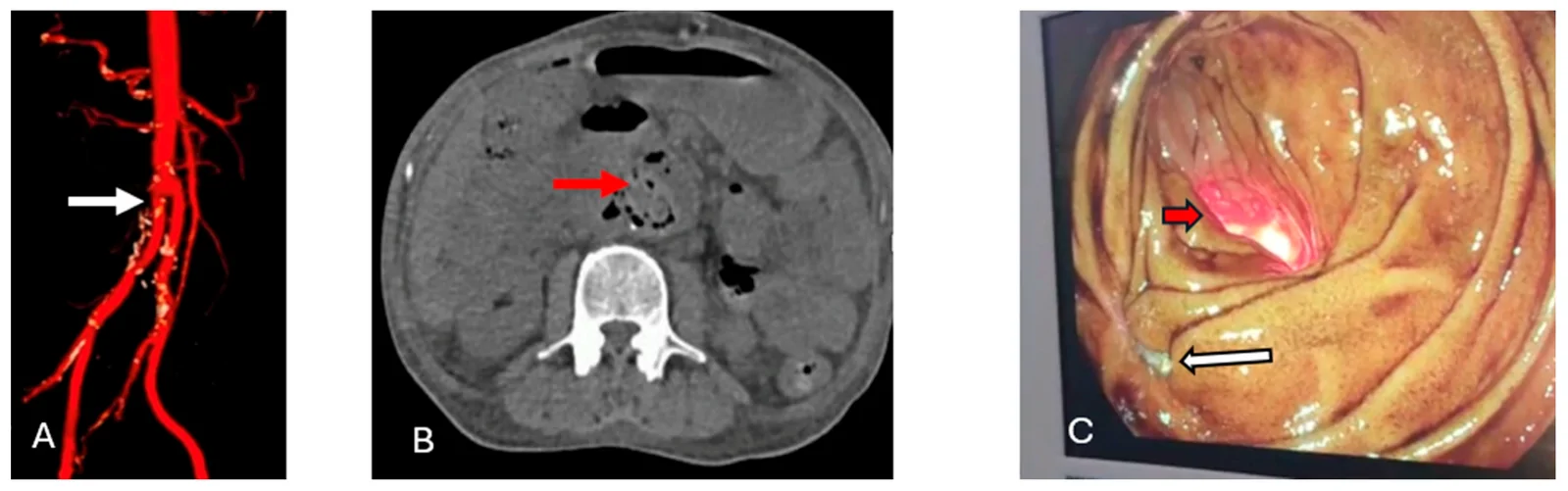
(Case 8): (**A**) Reconstructed CT scan with contrast (white arrow) indicating the graft following repair of rAAA with rifampin-bonded graft. (**B**) CT scan with contrast (red arrow) indicating air around the graft. (**C**) Intraoperative endoscopy: light of the endoscope in the third part of the duodenum (red arrow) and the tip of artery forceps (hemostats) introduced from the aneurysm sac to point to the fistula site (white arrow).

**Figure 6:**
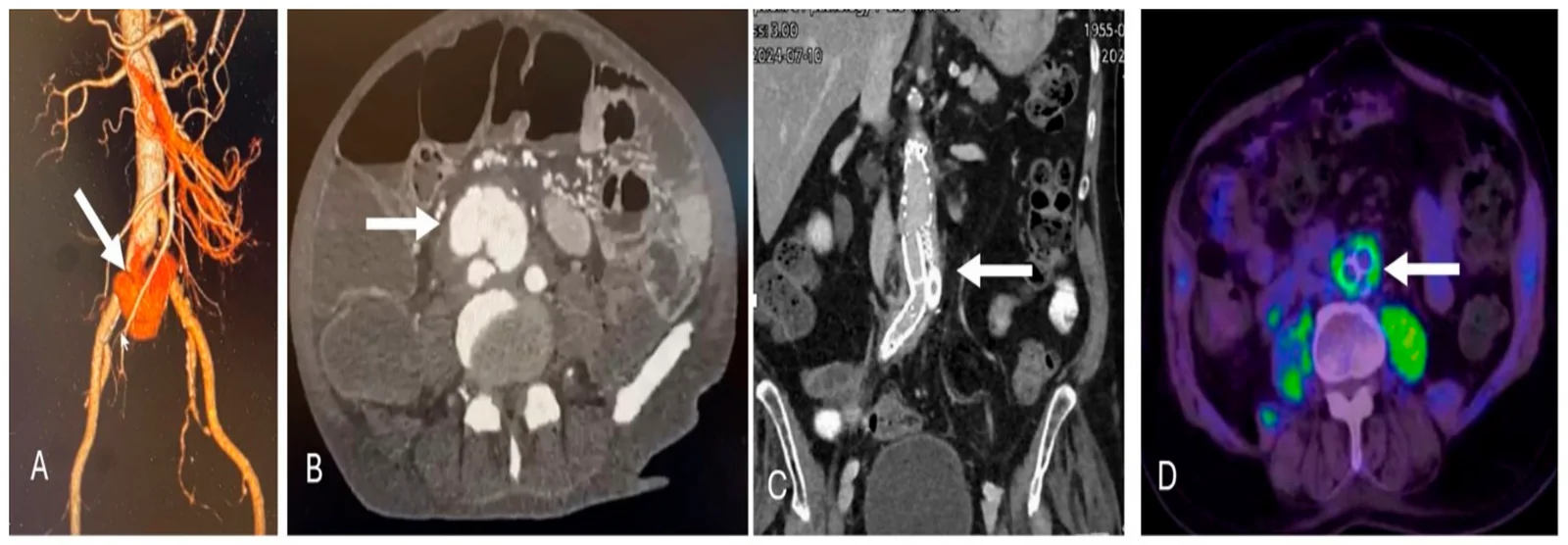
(Case 8): (**A**) Reconstructed CT scan with contrast, (white arrow) indicating the bovine pericardial bifurcated grafts with pseudoaneurysm. (**B**) CT scan with contrast (white arrow) showing the pseudoaneurysm. (**C**) CT scan with contrast (white arrow) showing EVAR stent within the bovine bifurcated grafts with complete resolution of the pseudoaneurysm (white arrow). (**D**) PET CT scan showing increased uptake of FDG (fluorodeoxyglucose) around an aortic graft, suggesting potential infection (white arrow).

AOF and ADF: Figure 1, Figure 2, Figure 3, Figure 4, Figure 5 and Figure 6 are examples of AOF cases number 3 and 4 and ADF cases number 5, 7 and 8. The main presentations were hypotension and upper GI bleeding. Six patients had ADFs, while four had AOFs. Blood cultures were positive in four cases, including Escherichia coli, Candida albicans, and Klebsiella. Blood and tissue culture results were given in Table 3.

Interventions: The mean time from the initial aortic operation and the presentation with secondary AEF was 8.05 years (0.2–25). Two patients underwent thoracic TEVAR. In the first case, hematemesis developed two weeks after sleeve gastrectomy. An endoscopically covered esophageal stent was placed, followed by massive bleeding from an AOF, requiring TEVAR. One year later, recurrent bleeding due to aortic stent infection occurred, and definitive treatment involved resection of the descending aorta with bovine pericardial patch reconstruction. In the second case, hematemesis occurred after a motor vehicle accident. A secondary AEF was confirmed by aortography and treated with TEVAR and a fully covered esophageal stent; the patient declined definitive surgery. The two secondary aortoduodenal patients underwent aortobifemoral bypass, and the second patient followed with EVAR. Both underwent an open repair, as did the four patients with primary AEF. Table 4 summarizes the surgical and endovascular interventions. Management of Aortic Graft Infection Collaboration (MAGIC) criteria for vascular graft and endograft infections [23] was applied and shown in Table 4.

**Aortoesophageal fistulas**

**Table 2:** Causes, types, times, intervention, outcomes and follow-up of the 4 aortoesophageal fistula (AOF) cases.

Patient Serial Number	Lesion and Cause	Time from Presentation to the Emergency Room to Vascular Intervention	Intervention	Outcome	Follow/up
1	Aortoesophageal fistula, secondary to a foreign body (chicken bone)	No vascular intervention patient was referred to gastroenterology as outpatient after 24 h following endoscopy patient was transferred to emergency room	None (quickly deteriorated)	Died from hypovolemic shock	-
2	Aortoesophageal fistula, secondary to a foreign body (fish bone)	Vascular consultation and surgery after >24 h	A left lateral thoracotomy was performed (operation not completed)	Died on the operating table with hypovolemic shock	-
3	Aortoesophageal fistula, secondary to the coarctation repair of the thoracic aorta done 25 years earlier	Vascular consultation and surgery in <6 h	Underwent TEVAR and IV Augmentin (amoxicillin/clavulanate potassium) for one week. Discharged on oral Septrin (cotrimoxazole) for 6 months	Responded well to treatment and remained alive on long-term antibiotics	7 y
4	Aortoesophageal fistula	The first operation three weeks from the primary surgery and Vascular consultation. Surgery in <1 h, the patient was taken from intervention radiology suite to hybrid operating room for the TEVAR The second operation patient was admitted with sepsis to medical intensive care unit and vascular consultation after patient started to bleed taken to surgery in <1 h	First operation attempt to control the bleeding by embolization using interlock coils field. Followed by endovascular intervention with implantation of 22 mm × 112 mm aortic stent (TEVAR using Valiant covered stent. Second operation done when the patient presented with massive hematemesis requiring surgical intervention (distal esophagectomy with removal of the endovascular stent and resection and replacement of the AEF site with reconstructed tube using 14 × 9 cm bovine pericardial graft through left thoraco-abdominal incision utilizing left cardiopulmonary bypass and distal perfusion. Reconstruction of the esophagus was done after 6 months with colonic interposition though the retrosternal route. Patient started with IV ceftriaxone. Antibiotic and antifungal medication changed over time based on the C&S. Currently not on antibiotics	Prolonged hospital stay and required total parenteral nutrition	8 y

**Aortoduodenal fistula (ADF) cases**

**Table 3:** Causes, types, times, intervention, outcomes and follow-up of the 6 aortoduodenal fistula (ADF) cases.

Patient Serial Number	Lesion and Cause	Time from Presentation to the Emergency Room to Vascular Intervention	Intervention	Outcome	Follow-Up
5	Aortoduodenal fistula	Vascular consultation and surgery in <6 h	Laparotomy with primary repair of the duodenum, excision of the old aortic graft, and in situ aortic reconstruction is performed with rifampicin-bonded synthetic grafts. The retroperitoneum closed with omental flaps to cover the graft	Died 2 weeks postoperatively due to sepsis and multiple organ failure	-
6	aortoduodenal fistula	Vascular consultation and surgery < 6 h	Primary duodenal repair, excision of the old graft, and in situ aortic reconstruction is performed with rifampicin-bonded synthetic grafts. The retroperitoneum closed with omental flaps to cover the graft. Six weeks on IV antibiotics and for 6 months on oral cotrimoxazole.	Died of cardiac causes unrelated to the fistula (5 years post-operation)	5 y
7	Aortoduodenal fistula		laparotomy with primary duodenal repair, explanation of a previously placed EVAR stent, and in situ aortic reconstruction is performed with rifampicin-bonded synthetic grafts. The retroperitoneum closed with omental flaps to cover the graft. 6 weeks on IV antibiotics and for 6 months on oral cotrimoxazole.	The patient recovered from sepsis	1 y
8	Aortoduodenal fistula		Primary duodenal repair, excision of the infected graft, and in situ aortoiliac reconstruction using a bovine pericardial graft. Six months later patient had recurrent infection underwent open surgical debridement without explanations and drain. Three month later had abdominal pain and CT showed pseudoaneurysm and EVAR within the bovine graft. Antibiotic and antifungal changes based on the culture and sensitivity. Patient started with IV ceftriaxone. Antibiotic and antifungal medication changed over time based on the C&S and Continue on IV Meropenem currently (18 months)	Recovered well and remained alive with no signs of sepsis	3 y
9	Aortoduodenal fistula (primary)		Primary repair of the duodenum along with aortic aneurysm repair using a rifampin-impregnated graft. Had postoperative bleeding from the left iliac anastomosis and managed with stenting. Had also leak from the duodenum which required rexploration and drainage.	Developed a duodenal leak and sepsis. Died within 9 weeks of surgery	-
10	aortoduodenal fistula (primary)		Primary repair of the duodenum and placement of a rifampin-impregnated graft with Primary closure of the retroperitoneum	Died 1 week postoperatively due to multiple organ failures	-

**Surgical and endovascular management**

**Table 4:** Surgical and endovascular management.

Variable	Open Surgery Group (*n* = 8)		Endovascular Group (*n* = 2)	
Age range (years)	30–80		30–40	
Sex (Male: Female)	8:0		1:1	
Type of Fistula	4 Secondary, 2 Primary		2 Secondary	
Fistula Location	6 Aortoduodenal, 2 Aortoesophageal		2 Aortoesophageal	
Positive perioperative blood culture	5/8 (62.5%)		1/2 (50%)	
Positive tissue culture	3/8 (37.5%)		1/2 (case #4 in the second stage operation after the removal of the endovascular stent and reconstruction of the thoracic aorta)	
Fungal organisms isolated	3 (Candida albicans)		1 (Candida + Klebsiella)	
Management of Aortic Graft Infection Collaboration (MAGIC) criteria for vascular graft and endograft infections and number of cases	Major Criteria: Clinical/surgical 7/8 Radiology 8/8 Laboratory 3/8	Minor criteria: Clinical/surgical 8/8 Radiology 8/8 Laboratory 5/8	Major Criteria: Clinical/surgical 1/2 Radiology 2/2 Laboratory 1/2	Minor criteria: Clinical/surgical 2/2 Radiology 2/2 Laboratory 2/2
Most Common Comorbidities	Hypertension, DM, renal failure and dyslipidemia		Smoking, obesity, and aortic coarctation.	
Surgical Intervention	Laparotomy, graft replacement		EVAR ± esophageal surgery	
Follow-up	0–5 years		7–8 years	
Outcomes				
Complications	Sepsis, organs failure, rebleeding		Esophageal stent bleeding	
30-Day mortality (*n* = 5)	5/8 (62.5%)		0/2 (0%)	
Long-term mortality (*n* = 1)	1/8		0/2	
Long-Term Survival (>1 Year)	2/8 (25%)		2/2 (100%)	

**Figures for AOFs:**

**Figures for ADFs:**

Outcomes and follow-up: The 30-day mortality was 30% (*n* = 3); one intraoperative death and two in-hospital deaths secondary to multisystem organ failure (all occurred in the open repair group). Additionally, there was one late in-hospital death due to sepsis and one late death on follow-up (one year) related to septic complications. One patient died after 5 years, which makes the overall AEF-related mortality 60% (*n* = 6).

**Figure 7:**
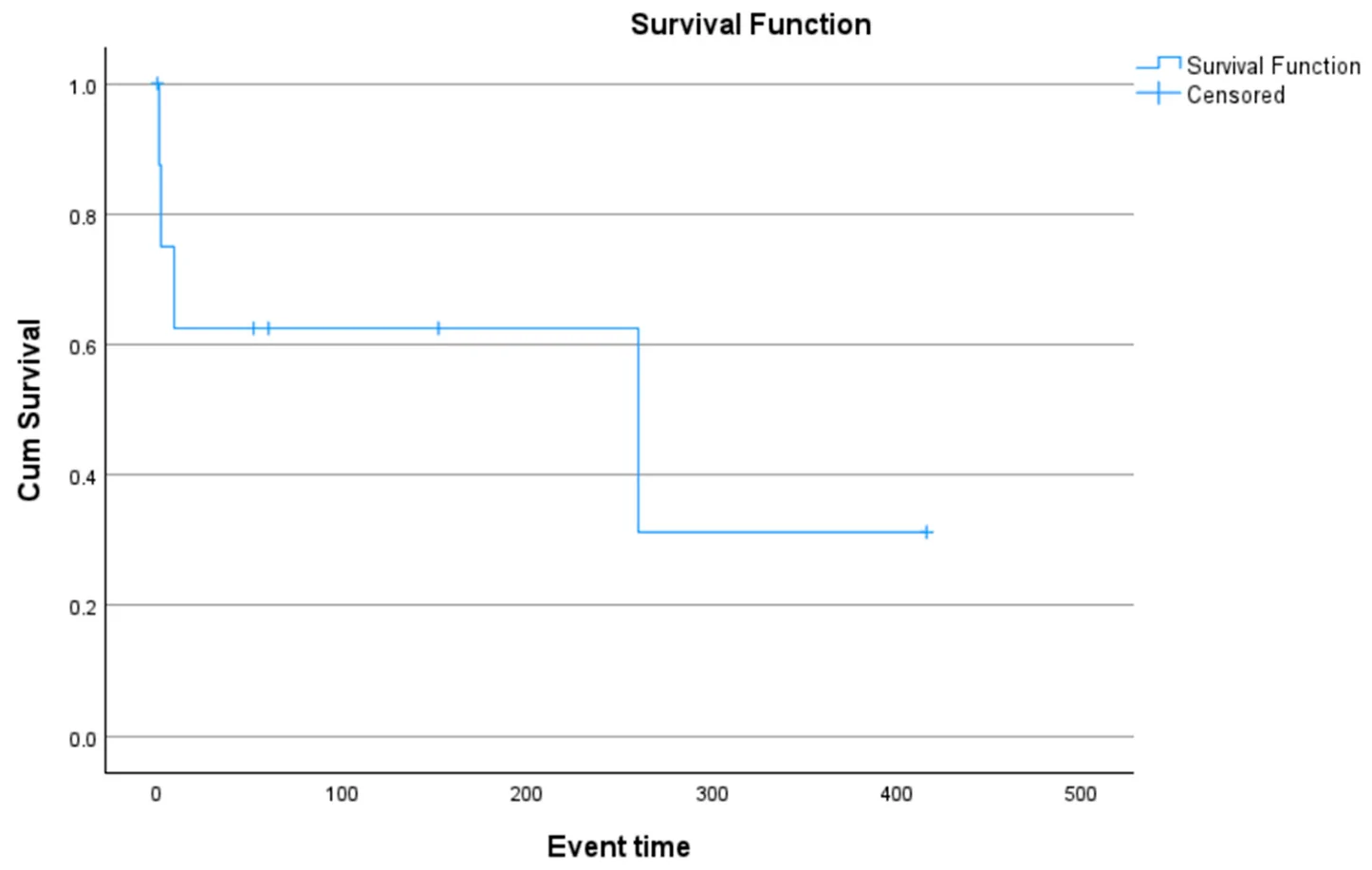
Kaplan–Meier survival curve.

Kaplan–Meier survival curve: Figure 7 shows an immediate drop in survival to 80% at week 0 due to 2 baseline events. The median survival time was 9 weeks (95% Confidence Intervals [CI] could not be reliably calculated due to the limited sample size). Following 9 weeks, the cumulative survival probability stabilized at 50% until dropping to 25% at 260 weeks. The final remaining patient was successfully censored at 416 weeks.

## 4. Discussion

This study on types of AEFs highlights the complexity and high mortality associated with this rare but life-threatening condition. In addition, we proposed a diagnostic algorithm. The present case series was completed in 2024, two years before the ESVS publication in 2026. However, our cases are in agreement with the new ESVS and the Delphi Consensus Document [24,25]. The guideline focused mainly on aortic pathology (aortic aneurysm or aortic graft), while our algorithm addresses type I (normal aorta) and type II (normal GIT). Regarding partial and complete erosion, it is consistent with our cases. In the algorithm, we subdivided type II into three subgroups (SI for partial thickness erosion of the wall of the aorta or GIT, SII for full thickness erosion of the wall of the aorta or GIT, and SIII for full thickness erosion of both walls).

AEFs have an approximate 100% mortality rate if left untreated, but if treated within 60 days, mortality decreases to 50% [26,27,28]. In the present study, there was no AEF related to malignancy. In the literature, AEF due to malignancy are mostly case reports or case series, and most of the systematic reviews have a small percentage of malignancy-related AEFs [7,8]. The patient cohort was predominantly male (90%), with a mean age of 56 years, consistent with the existing literature [2,4]. Blood cultures were positive in 40% of cases, with Candida species being the most frequently isolated organism. This suggests that fungal infection, particularly Candida species, significantly contributes to graft-related complications, as reported in the literature [13,28,29].

Although AOFs and ADFs are distinct entities, this study is insufficient to demonstrate substantial similarities or differences within the context of AEFs; therefore, multicenter, large-sample-size studies including both entities are warranted. Prior data focused on AEF etiology [30,31]. This is because the pathology and presentation are relatively comparable in its variants (AOFs and ADFs), apart from the anatomical location [30,31]. However, the complexity of intervention and mortality rate could differ between the two entities.

While bleeding risk is the dominant acute concern in patients with AEFs, there are differences in procedural complexity based on its etiology. For instance, the foreign body has the same risk of AEF-related bleeding as with the other underlying etiologies. However, the intervention’s complexity may differ. The occurrence of complications in the long term, such as bleeding or infection, is the same regardless of the etiology; however, the long-term infection rate is higher in patients with endovascular repair for secondary AEFs compared with primary AEFs. Long-term survival is frequently limited by persistent or recurrent infection [12,13,27].

The initial diagnostic test for AEFs should be CT angiography (CTA), which has high sensitivity and specificity [32,33]. Nuclear scans are also helpful in slow-progressing pathology [27].

Surgical intervention was attempted in eight of the ten patients. Two patients (cases 1 and 2) died before definitive surgical repair could be undertaken. Among those who underwent surgery, the most common approach was open repair with graft excision and replacement using rifampin-impregnated or bovine pericardial grafts [21]. One patient underwent TEVAR, and another underwent a complex esophageal resection with colon interposition. Bovine pericardial grafts were used in one AOF and one ADF in our study; they may help reduce the risk of infection and dependence on long-term antibiotics, emphasizing the importance of individualized treatment to optimize survival [34,35]. However, more studies are needed to support this finding. These advanced techniques reflect a growing trend toward multidisciplinary and staged surgical management in complex AEF cases [22].

Despite aggressive surgical strategies, the overall mortality rate was 60%, with most deaths occurring in the immediate postoperative period or due to complications such as shock, multiple organ failure, or persistent infection [3,21]. Survival was achieved in 4 patients, all of whom underwent timely surgical intervention. These survivors were followed for periods ranging from one to eight years, indicating that long-term survival is achievable with effective source control and comprehensive perioperative care. Notably, even patients with severe sepsis and positive fungal cultures (cases 6 and 10) survived when treated with extensive surgical reconstruction and long-term antimicrobial therapy [13,22].

Historical development of endovascular treatment has transformed AEF management. Before the 1990s, the treatment was exclusively open surgical intervention: technically demanding and frequently unsuccessful due to patients’ general condition at the time of presentation (massive bleed, shock, sepsis and multiple comorbidities). The introduction of endovascular techniques shifted treatment from high-risk emergency open surgery to endovascular surgery capable of immediate hemorrhage control by excluding the aortic defect. In the same way, the introduction of gastrointestinal track stenting reduces the amount of contamination from the GIT. Although not usually curative by itself, endovascular stenting has improved survival by serving as an effective bridge to definitive surgical and multidisciplinary treatment [36,37,38,39].

Elliott et al. [40] first highlighted the diagnostic and therapeutic challenges of AEFs in the context of aortic grafting. They introduced the crucial conceptual distinction between true AEFs and paraprosthetic enteric fistulas. Building on this concept, subsequent authors, notably Szilagyi and later Bunt, proposed a subtype classification of secondary AEFs into Type I, characterized by a direct graft–enteric communication often associated with suture-line disruption or pseudoaneurysm formation, and Type II, involving paraprosthetic enteric erosion without a true fistula, frequently presenting with sepsis or chronic infection rather than acute hemorrhage [41,42]. With the advent of EVAR, additional subcategories of secondary AEFs have been recognized, including fistulas occurring after stent–graft placement, which may result from graft migration, endoleak-associated infection, or mechanical erosion [43]. Our diagnostic proposal is to combine all these classifications to reduce confusion arising from the different classification formats while preserving the fundamental primary-versus-secondary framework.

**Figure 8:**
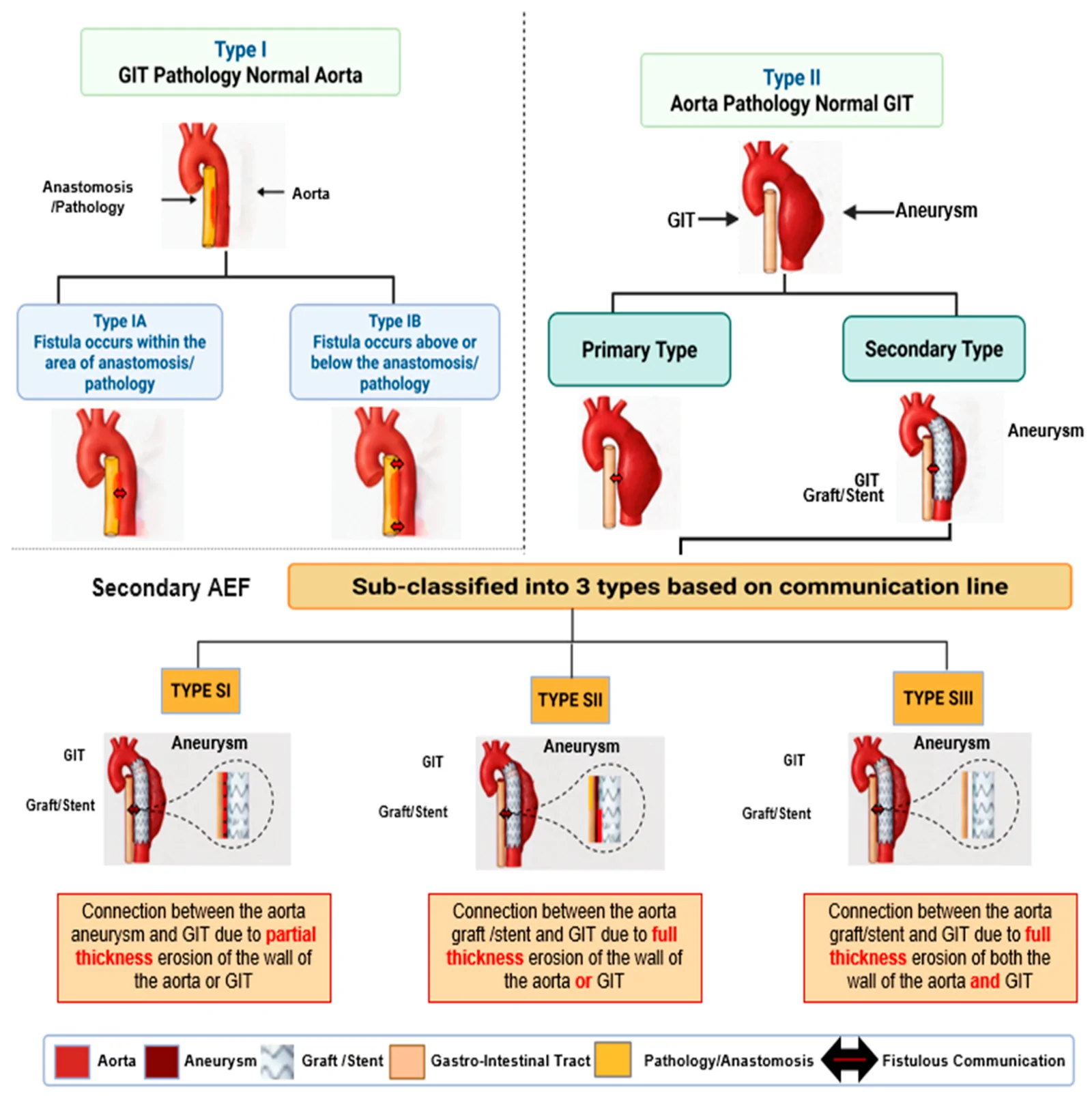
Proposed classification for aortoenteric fistula (AEF) pathology and diagnosis. GIT: gastrointestinal tract; pathology: benign (inflammation, infection, etc. or malignant (primary or secondary)).

Diagnostic algorithm: The proposed diagnostic classification of AEFs could improve the understanding of location and relation to the aorta, and to consider combined pathology on both sides (GI tract and Aorta) (Figure 8). This algorithm could guide decision-makers for timely and appropriately applied management; however, it is not a recommended pathway but a potential option that requires further exploration and validation. From a GIT pathology perspective, it could be an inflammatory process, causing chemical erosion, malignancy, or the impact of a primary intervention (such as a stent). From the aortic side, it could be due to mechanical pulsation and pressure or gradual aortic graft erosion. Also, infection of the aortic graft can cause inflammation and erosion [38]. The algorithm development was based on our department’s experience and learning curve over the years, including local and international consultations with vascular surgeons and vascular clinical researchers.

AEF preventive measures [27,44,45,46,47]:

- Patient education, including instructions for atherosclerosis risk factors, timely colonoscopy, and adherence to follow-up post-interventions for a long time.
- A one-time screening ultrasound of the aorta has been recommended for older male patients (65–75 years) who have ever smoked in their lives.
- During open surgical repair of an AAA, the surgeon must ensure that the aortic graft material is sufficiently covered with a layer of tissue. This can be achieved by reapproximating the aneurysmal sac around the graft or by creating an omental flap or vascularized pedicle to buttress the repair. In aortobifemoral bypass, the graft must be covered with omentum or a vascularized pedicle.
- AEF repair survivors must undergo regular aortic screening examinations for the rest of their lives.
- A high index of suspicion is strongly recommended to avoid delayed diagnosis of AEFs and its subsequent complications and poor outcomes in both young and older populations.

**Figure 9:**
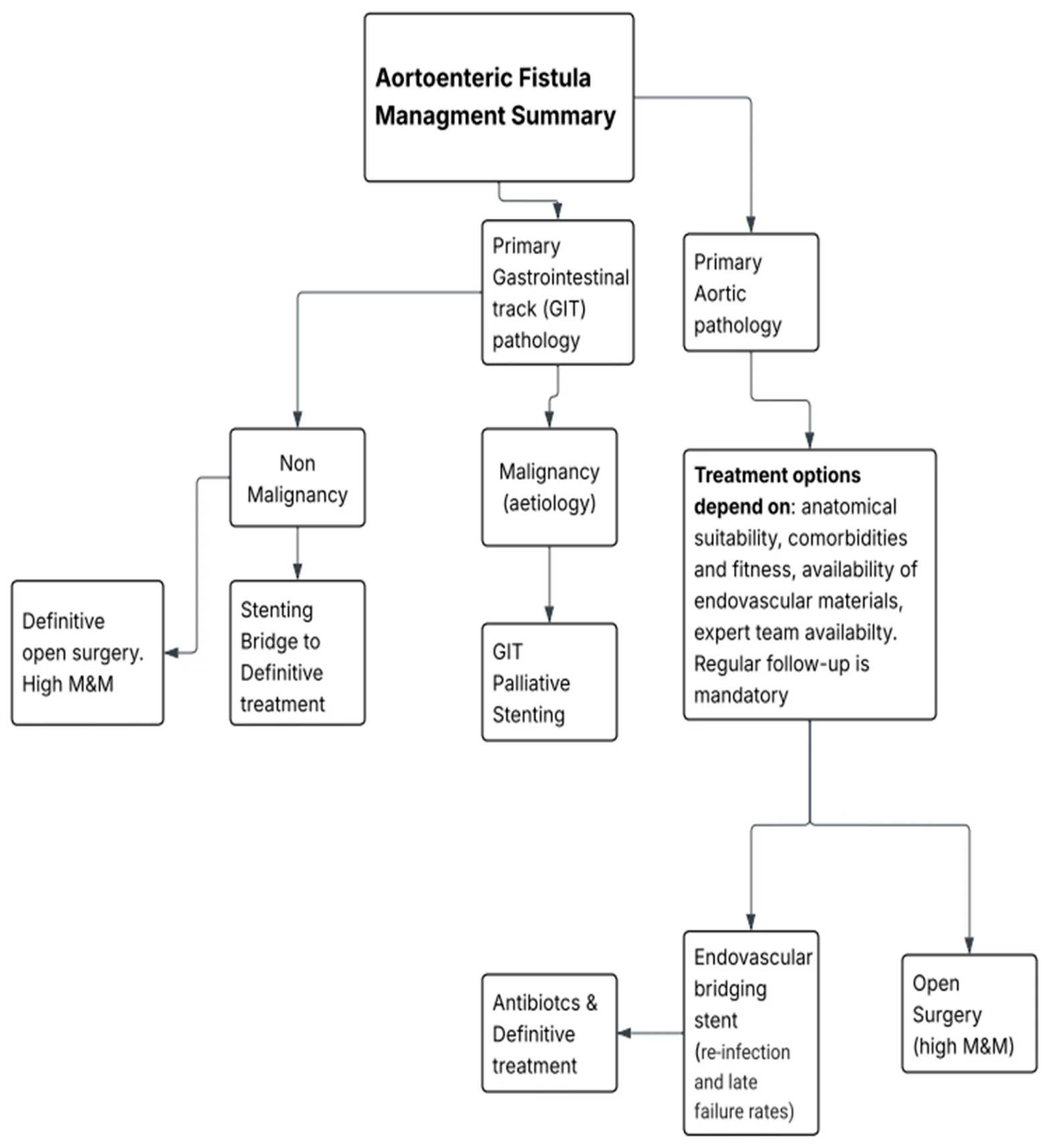
Management of AEFs (M&M: morbidity and mortality).

Management of AEFs:

We summarized the management options for AEFs as shown in Figure 9, which reveals the indications for OSR and endovascular repair. This case series highlights the high risk and clinical complexity associated with AEFs. Early recognition, prompt surgical intervention, and aggressive infection control are essential for improving outcomes. While open repair remains the standard in most cases, endovascular and reconstructive techniques provide viable alternatives or adjuncts in selected patients [21,22,27]. The endovascular approach, for patients not stable enough for open surgery, has an in-hospital mortality of 7%, whereas open repair has a 34% mortality [21,27]. The rate of late sepsis is lower with OSR than with endovascular repair (19% vs. 42%) [21]. The presence of fungal or polymicrobial infection significantly impacts prognosis but does not affect survival when appropriately and timely managed [13].

In general, the management of AEFs depends on a number of factors. (1) The patient’s hemodynamic stability. (2) The facility and the experience of the multidisciplinary team managing the patient. (3) The first stage or the bridging stage. Management of the aortic pathology to control the bleed in the acute setting by endovascular or open surgery, depending on the patient’s general condition, and to control the contamination from the GIT by cover stent, diversion or resection. (4) When the patient’s condition becomes stable, address the aortic pathology by open surgery and gastrointestinal reconstruction. (5) In case of malignancy: if the patient needs palliative care, stage one may be the only option. Conservative management can be a valid option in case of malignancy: palliative care and empirical broad-spectrum antibiotics and antifungal therapy initially, modified based on the blood/tissue culture in case of high-risk patients. There is no clear consensus on the duration of antibiotics, but it needs to be according to clinical and laboratory findings.

Limitations: The retrospective nature of the study, the small sample size, and the single-center design are limitations. In this study, AEF terminology was used to define any location in the aorta and GIT unless it was specified, such as AOF or ADF. Further research, including multicenter registries and prospective studies, is warranted to develop standardized guidelines for optimal AEF management and to identify factors that predict survival in this critically ill patient population. However, as shown in Table 5, publications over the last two decades mainly included single case reports, small case series, and a few retrospective studies [1,9,10,11,12,15,18,19,20,26,28,33,39,48,49,50,51,52]. All case series, like ours, are limited to almost 10 cases, whereas multicenter studies range from 29 to 47 cases. Moreover, the duration of studies is very long, indicating the rarity of this condition.

## 5. Conclusions

This descriptive case series showed that AEFs are an uncommon and fatal condition that requires a high index of suspicion and timely, structured management and follow-up. The initial endovascular approach serves as an effective bridge to stabilize hemodynamics; subsequent definitive surgical repair is necessary. Post-repair infection and the need for lifelong antibiotics may be reduced by using bovine pericardial grafts; however, the number of cases remained low. There is a critical need for larger-scale, collaborative research efforts to address the diagnostic complexity and high mortality associated with AEFs. This will enhance our understanding of the diverse clinical presentations and treatment responses and help develop evidence-based, standardized protocols for diagnosis, surgical intervention, infection control, and long-term follow-up.

## Figures and Tables

**Table 1:** Demographic, presentations, fistula class, times and preoperative investigations of the 10 AEFs.

Patient Serial Number	Sex/Age	Presentation and Class *	Interval from Previous Aortic Surgery to Presentation (Years)	Comorbidities	Preoperative Investigation/ Consultations
1	Young Male (1)	upper GI bleeding and chest pain. Negative blood culture. **Type 1A**	-	None	CT, Endoscopy/ Gastroenterology
2	Young Male (2)	upper GI bleeding, chest pain, and hypotension. **Type 1A**	-	Smoking, Hypertension	CT, Endoscopy/ Gastroenterology, Acute Care Surgery/Vascular Surgery
3	Young Male (1)	chest pain, syncope, upper GI bleeding, Negative blood culture. **Type II (SII)**	25	Smoking, Coarctication of thoracic aorta	CT, Endoscopy/ Gastroenterology, Acute Care Surgery/Trauma Surgery/Vascular Surgery
4	Young Female (1)	abdominal pain, upper GI bleeding, melena, hypotension, and sepsis. Positive blood culture (Klebsiella pneumoniae and Candida). **Type IA** (leak from distal esophagus following sleeve gastrectomy)	Two presentation; the first two week after complicated sleeve gastrectomy and the second after one year from the endovascular stent	Obesity	CT, Endoscopy/ Gastroenterology, Acute Care Surgery/Bariatric Surgery/Intervention Radiology/Vascular Surgery/Cardiac Surgery
5	Old Male (3)	upper GI bleeding, melena, and hypotension. Negative blood cultures. **Type II (SII)**	4	Smoking, Hypertension, Peripheral arterial disease	CT, Endoscopy/Gastroenterology, Acute Care Surgery/Vascular Surgery
6	Old Male (4)	upper GI bleeding, melena, and hypotension. **Type II (SII)**	6	Smoking, Hypertension	CT, Endoscopy/Gastroenterology, Acute Care Surgery/Vascular Surgery
7	Old Male (3)	abdominal pain, melena, and hypotension. Positive blood cultures (Escherichia coli). **Type II (SII)**	1	Smoking, Hypertension, Dyslipidemia, Chronic kidney disease on hemodialysis	CT, Endoscopy, Gastroenterology, and Vascular Surgery
8	Old Male (4)	history of sepsis presented with abdominal pain, melena, and hypotension. Positive blood cultures (Candida albicans). **Type II (SII)**	2/12	Hypertension, dyslipidemia	CT, Endoscopy, Gastroenterology, Acute Care Surgery and Vascular Surgery
9	Old Male (4)	abdominal pain, melena, and hypotension. Positive blood cultures (Klebsiella and Candida). **Type II (primary)**	-	Multiple Cerebral infarcts, DM, Hypertension, Dyslipidemia, Post-renal transplant	CT, Endoscopy, Gastroenterology, and Vascular Surgery
10	Old Male (3)	abdominal pain, melena, and hypotension. Negative blood culture). **Type II (primary)**	-	Smoking, Coronary artery disease, CABG, DM, Hypertension	CT, Endoscopy, Gastroenterology, Acute Care Surgery and Vascular Surgery

For de-identification: young age: (1) 30–40 and (2) 41–54 years old. * The class was given based on the proposed diagnostic algorithm. Old age: (3) 55–65 and (4) 66–80 years. The bold in the third column indicates the classification used according to the proposed algorithm.

**Table 5:** Examples of recently published English-language literature on aortoenteric fistulas.

Author and Year	Study Type	PAEF or SAEF	Type of AEF	Management
Luo et al. 2021 [1]	Case series single center	- 5 PAEF - 4 SAEF	- 4 ADF - 5 AEF	- 3 EVAR - 3 ORS - 2 EVAR and ORS - 1 refused
Thelin et al. 2015 [9]	Case report	- 1 SAEF	- ADF	- EVAR
Chick et al. 2017 [10]	Case report	- 1 PAEF - 2 SAEF	- 2 ADF - 1 AOF	- EVAR
Partovi et al. 2018 [11]	Case series single center (*n* = 4)	- 3 PAEF - 1 SAEF	- 3 AOF - 1 AJF	- 1 OSR - 2 EVAR - 1 not mentioned
Kieffer et al. 2003 [15]	Case series single center (*n* = 9) over 14 years	- 2 PAEF - 7 SAEF	- 9 AOF	- 6 OSR - 2 died before assessment - 1 refused
Zhong et al. 2022 [18]	Case report	- 1 SAEF	- AOF	- EVAR
Armstrong et al. 2005 [19]	Retrospective analysis (*n* = 29) over 13 years	- 29 SAEF	- ADF	- OSR
Deijen et al. 2016 [20]	Retrospective analysis (*n* = 32 pts **) over 19 years	- 5 PAEF - 27 SAEF	- 26 ADF - 2 AOF - 4 other AEFs	- 10 EAR - 10 ISR - 5 EVAR - 5 No intervention (died) - others (not clear)
Gregg et al. 2024 [26]	Case report	- 2 PAEF	- ADF	- OSR
Batt et al. 2017 [28]	Retrospective multicenter analysis (*n* = 37) over 8 years	- 37 SAEF	- AEF	- EAR: 9, ISR: 25 - EVAR:3
Hughes et al. 2007 [33]	Retrospective single center analysis (*n* = 10) over 20 years	- 2 PAEF - 8 SAEF	- 7 ADF - 3 AEF	- Not given
Papacharalambous et al. 2007 [45]	Case report	- 1 PAEF	- ADF	- EVAR
Hansen et al. 2017 [49]	Case report	1 SAEF	- 1 ADF	Recurrence twice. Survived 12 years post-non-curative surgery
Skourtis et al. 2010 [12]	Case report	1 PAEF	- 1 ADF	- intestinal repair - OSR
Georgeades et al. 2021 [50]	Case report	1 PAEF	- 1 ADF	EVAR followed by ORS and duodenal surgery
Oikonomou et al. 2022 [51]	Retrospective single center (*n* = 23) over 13 years	23 SAEF	- 21 ADF - 2 AEF	- 15 ISR, 2 EAR, 5 OSR - 2 EVAR
Wu et al. 2025 [52]	Retrospective single center (*n* = 10) over 2 years	- 2 PAEF - 8 SAEF	- 7 ADF - 3 AJF	- 10 ISR
Narayanan et al. 2023 [39]	Retrospective multicenter (*n* = 47) over 17 years	- 47 SAEF	- AEF	- 22 EAR, 4 ISR, 4 OSR - 9 EVAR only - 6 EVAR as a bridge to OSR - 8 none
This paper	Case series single center (*n* = 10) over 16 years	- 4 PAEF - 6 SAEF	- 4 AOF - 6 ADF	- 8 OSR - 2 EVAR

AEF: aortoenteric fistula in general or unspecified; PAEF: primary lesion; SAEF: secondary lesion. In Situ Reconstruction (ISR) rebuilds the graft in its original location using infection-resistant materials. Extra-Anatomic Reconstruction (EAR): reroutes the graft away from the infected site. AJF: aorto-jejunal fistula; ** 2 iliac fistulas not included. EVAR: endovascular aortic repair; ADF: aortoduodenal fistula; AOF: aortoesophageal fistula.

## Data Availability

The original contributions presented in this study are included in the article. Further inquiries can be directed to the corresponding author.

## Abbreviations

AAA	Abdominal aortic aneurysm
TEVAR	Transcutaneous endovascular aortic repair
AEF	Aortoenteric fistula
GI	Gastrointestinal
ADF	Aortoduodenal fistula
AOF	Aortoesophageal fistula
